# Accumulation Pattern and Risk Assessment of Potentially Toxic Elements in Permafrost-Affected Agricultural Soils in Northeast China

**DOI:** 10.3390/toxics11070632

**Published:** 2023-07-21

**Authors:** Junbo Yu, Chuanfang Zhou, Ke Yang, Qifa Sun, Qipeng Zhang, Zhiwei Yang, Yangyang Chen

**Affiliations:** 1 Harbin Center of Natural Resources Integrated Survey, China Geological Survey, Harbin 150086, China; yujunbo@mail.cgs.gov.cn (J.Y.);; 2 Institute of Geophysical and Geochemical Exploration, Chinese Academy of Geological Sciences, Langfang 065000, China; 3 Shenyang Center of Geological Survey, CGS, Shenyang 110034, China

**Keywords:** agricultural soil, permafrost, potentially toxic elements, potential ecological risk, human health risk

## Abstract

The accumulation of potentially toxic elements (PTEs) in agricultural soils is of particular concern in China, while its status, ecological risks, and human health hazards have been little studied in the permafrost areas of Northeast China. In this study, 75 agricultural soil samples (0–20 cm) were collected from the Arctic Village, Mo’he City, in the northernmost part of China. The average concentration (mean ± standard deviation) of As, Cd, Cr, Cu, Hg, Ni, Pb, and Zn were 12.11 ± 3.66 mg/kg, 0.11 ± 0.08 mg/kg, 52.50 ± 8.83 mg/kg, 12.08 ± 5.12 mg/kg, 0.05 ± 0.02 mg/kg, 14.90 ± 5.35 mg/kg, 22.38 ± 3.04 mg/kg, and 68.07 ± 22.71 mg/kg, respectively. Correlation analysis, cluster analysis, and principal component analysis indicated that As, Cu, Ni, and Zn likely originated from geogenic processes, Hg and Pb from long-range atmospheric transport, Cd from planting activities, and Cr from Holocene alluvium. The geo-accumulation index and enrichment factor showed that As, Cd, Hg, and Zn are enriched in soils. The Nemerow pollution index showed that 66.67%, 24%, and 1.33% of soil samples were in slight, moderate, and heavy pollution levels, respectively, with Hg being the most important element affecting the comprehensive pollution index. The potential ecological risk index showed that 48.00% and 1.33% of soil samples were in the moderate ecological risk and high potential ecological risk levels, respectively. The non-carcinogenic and carcinogenic human health risk index for adults and children were both less than 1, which was within the acceptable range. This study revealed the accumulation pattern of PTEs in agricultural soils of permafrost regions and provided a scientific basis for research on ecological security and human health.

## 1. Introduction

Heavy metal(loid)s (As, Cd, Cr, Cu, Hg, Ni, Pb, and Zn) are considered potentially toxic elements (PTEs) due to their high toxicity, long residence time, and persistent bioavailability [1,2]. The presence of PTEs in agricultural soils could come from a variety of sources, including the weathering of parent materials, mining, smelting, traffic emissions, application of chemical fertilizers, or the disposal of domestic waste [3,4,5,6].

The 2014 official bulletin, Reports on China’s Soil Pollution Survey, also concluded that 19.4% of metal(loid) concentrations in agricultural soils across China exceeded the guideline value [7,8]. Of the contaminated soil samples, 82% contained toxic inorganic pollutants, the most common being PTEs such as cadmium, mercury, arsenic, chromium, and lead, which can cause chronic health problems [9,10,11,12,13,14,15,16]. Given the high PTE concentrations in agricultural soils, China faces the challenge of controlling soil contamination to ensure food safety and ecological security [17].

Permafrost is ground material that remains at or below 0 °C for 2 or more consecutive years and is widespread at high latitudes and elevations [18]. Due to the cold air temperature of permafrost regions, atmospheric deposition rates in these regions are high, and various pollutants are deposited and accumulated in these regions [19,20,21,22,23]. As the climate warms, permafrost degradation may result in the release of these pollutants to the atmosphere in gaseous form/bound to organic particles or export in liquid form to rivers, further threatening ecosystems and human health [24,25].

In addition, rising air and soil temperatures favor population and agriculture expansion in cold regions [26,27]. The northeast plain is one of the main grain-producing regions in China, as well as the second-largest extent of permafrost and the most important region of latitudinal permafrost in China [28,29]. However, the existing studies on PTEs in permafrost soils in China mainly focused on the Qinghai–Tibet Plateau [30,31,32,33,34], while studies in northeast China are rare. Therefore, there is no comprehensive information on PTE contamination in permafrost-affected agricultural soils in northeast China and its effects on the ecosystem and human health.

Therefore, the objective of this study was to (1) investigate the accumulation status and contamination level of eight PTEs (As, Cd, Cr, Cu, Hg, Ni, Pb, and Zn) in permafrost-affected agricultural soils; (2) evaluate the potential sources of these PTEs in agricultural soils; and (3) assess the ecological safety and risk to human health from the PTEs in agricultural soils. The results will enhance understanding of the sources, accumulation patterns, potential environmental risks and human health risks of PTEs in the high latitude permafrost regions.

## 2. Materials and Methods

### 2.1. Study Area

**Figure 1:**
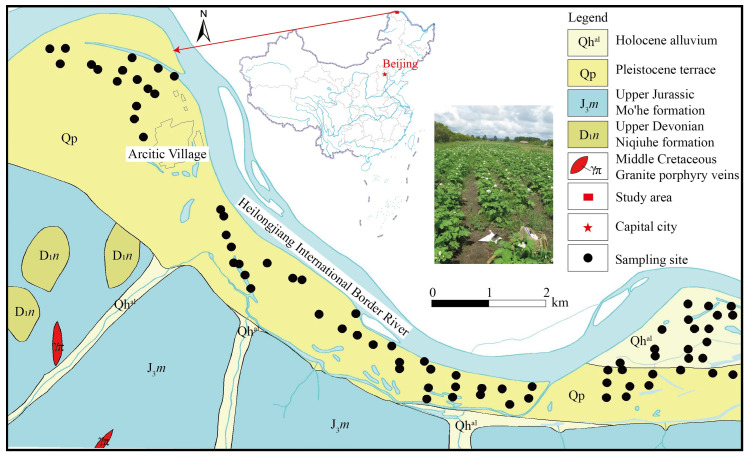
Location and geology of the study area and spatial distribution of the sampling sites. Holocene alluvium: black clay, crushed stone, gravel, fine sand, silt; Pleistocene terrace: yellow gray sub-clay, light yellow fine medium sand; Upper Jurassic, Mo’he formation: The upper part is interlayered with sandstone and silty mudstone, and the lower part is dominated by sandstone with conglomerate; Upper Devonian, Niqiuhe formation: Argillaceous siltstone interspersed with limestone; Middle Cretaceous, Granite porphyry veins.

The study area is located in the Arctic Village, Mo’he City, which is in the northernmost permafrost regions of China, with a total area of about 16 km^2^, spanning the latitude 53.450~53.558° N, longitude 122.351~122.358° E, and elevation 285~300 m a.s.l. (Figure 1). The permafrost layer is 2–3.5 m deep and 3–6 m thick.

The Heilongjiang International Border River runs to the north, while the Da Xing’anling Mountains lie to the south. The study area has a cold temperate continental monsoon climate with an average annual temperature of −4.1 °C, an average annual rainfall of 430.6 mm, and an annual evaporation of 886 mm. The stratigraphy of the cropland is mainly Holocene alluvium and Pleistocene terrace. The main crop varieties are potatoes, soybeans and Chinese herbs.

### 2.2. Soil Sampling

Seventy-five soil samples were collected in September 2020. All sampling points were located using a global positioning system (GPS). The sampling density is 8 points per km^2^ in a 500 m × 250 m grid. Avoiding non-representative areas such as ditches, forest strips, field margins, and roadsides, each sample was dug and mixed in 6 pits in the “S” direction with a depth of 0–20 cm, and the average weight of the collected samples was about 500 g. After collection, the samples were placed in cloth bags, numbered, sealed, weighed, and dried in a clean and airy place. Then, the air-dried samples were sieved through a 10-mesh nylon sieve to remove plant roots, gravel and other debris. Finally, the samples were divided into equal parts and sent to the laboratory for analysis.

### 2.3. Analytical Methods

Soil samples were analyzed for As, Cd, Cr, Cu, Hg, Ni, Pb, Zn, Sc, and pH by the laboratory of the Harbin Center of Natural Resources Integrated Survey. The analytical methods and detection limits are listed in Table 1. Detailed descriptions of the methods can be found in Zhang et al. [35], while the quality assessment is described in detail by Li et al. [36]. Internal and external controls were performed during routine analysis to verify accuracy and precision. Briefly, certified reference materials (CRMs) and blind reference materials (BRMs) were analyzed simultaneously with the samples to assess the accuracy and precision of the sample analysis [36]. The accuracy and precision requirements were listed in Table 2, and for pH, the relative deviation (RD) between the sample and CRMs should be met with |ΔpH| ≤ 0.1. The accuracy and precision of all elements in all samples met the analytical requirements developed as part of the NMPRGS/NGSLQ project [37].

### 2.4. Data Analysis

The calculation of indices characterized by different features helps to find or create the right theoretical basis for a proper interpretation of soil conditions. In this work, the geo-accumulation index (*I_geo_*), the enrichment factor (*EF*), the Nemerow pollution index (*NPI*), the coefficient of potential ecological risk of a single PTE (*E_r_*), and the potential ecological risk index (*PERI*) were used to comprehensively evaluate the pollution status of PTEs (As, Cd, Cr, Cu, Hg, Ni, Pb, Zn). The human health index (*HI*) was used to evaluate non-carcinogen and carcinogen risks in permafrost-affected agricultural soils in northeast China. The classification systems *I_geo_*, *EF*, *NPI*, *E_r_*, and *PERI* are listed in Table 3.

#### 2.4.1. Geo-Accumulation Index (I_geo_)

Muller [38] proposed the concept of the geo-accumulation index (*I_geo_*), which can be used to estimate changes in PTEs in the soil to assess the impact of human activities. This index can be calculated as Equation (1):

(1) $$I_{geo}={log}_{2}[\frac{c_{i}}{1.5\times B_{i}}]$$

where *C_i_* and *B_i_* are measured and background PTE concentrations in soils. The coefficient, 1.5, is used to minimize possible variations due to lithogenic variations. The background values for As, Cd, Cr, Cu, Hg, Ni, Pb, and Zn obtained from Heilongjiang Province are 8.60, 0.09, 54, 19, 0.022, 23, 22, and 56 mg/kg, respectively [39]. The pollution classification scheme is shown in Table 3.

#### 2.4.2. Enrichment Factor (EF)

The EF is specified by standardizing a tested element against a reference element with a low variability of occurrence [40,41]. Reference elements are usually those for which the concentration in the sample medium will practically exclusively be influenced by crustal sources. In this study, Sc was selected as the reference element, due to its conservative geochemical property, at a concentration of 9.8 mg/kg. This index can be calculated as Equation (2):

(2) $$EF=\frac{{\left(C_{i}/C_{r}\right)}_{s}}{{\left(C_{i}/C_{r}\right)}_{b}}$$

where *C_i_* and *C_r_* are the measured and reference elements, and s and b are the sample and background. The pollution classification scheme is shown in Table 3.

#### 2.4.3. Nemerow Pollution Index (NPI)

The *NPI* is used to assess the overall situation of PTEs in soils [42,43]. This index considers not only the impact of PTEs with high concentrations on the environment but also the impact of individual PTE on environmental quality by analyzing their mean value. This index can be calculated as Equations (3) and (4):

(3) $$PI=\frac{C_{s}^{i}}{C_{n}^{i}}$$

(4) $$NPI=\sqrt[{2}]{\frac{{PI}_{ave}^{2}+{PI}_{max}^{2}}{2}}$$

where *PI* is the pollution index of the PTE element *i* in the soil, $C_{s}^{i}$ is the concentration of PTE *i* in the sample, and $C_{n}^{i}$ is the value of background concentration in Heilongjiang Province. *NPI* is the comprehensive pollution index of the sampling site; *PI* is the one factor index evaluation value of PTE_i_; *PI_max_* is the maximum value of *PI*; and *PI_ave_* is the average value of *PI*. The pollution classification scheme is shown in Table 3.

#### 2.4.4. Potential Ecological Risk Index (*PERI*)

PTEs pose potential ecological risks to soil systems. The potential ecological risk index (*PERI*) represents the sensitivity of a biological community to contaminants and illustrates the resulting potential ecological risk [44]. This index can be calculated as Equation (5):

(5) $$PERI=\sum_{i=1}^{n}E_{r}^{i}=\sum_{i=1}^{n}\left(T_{r}^{i}\times C_{f}^{i}\right)=\sum_{i=1}^{n}\left(T_{r}^{i}\times \frac{C_{s}^{i}}{C_{n}^{i}}\right)$$

where *PERI* is the index of potential ecological risk; $E_{r}^{i}$ is the coefficient of potential ecological risk of a single PTE; and $T_{r}^{i}$ is the toxicity coefficient of the single PTE. The toxicity coefficients of the PTEs are as follows [44]: Hg = 40, Cd = 30, As = 10, Cu = Ni = Pb = 5, Cr = 2, and Zn = 1. $C_{s}^{i}$ and $C_{n}^{i}$ are measured- and background-PTE concentrations in soils. The pollution classification scheme is shown in Table 3.

#### 2.4.5. Human Health Risk Index (HI)

The health risk assessment model published by USEPA [45] was used to evaluate human health risks. The assessment steps included exposure calculation and risk characterization. PTEs in soil are absorbed by humans in three ways: direct oral ingestion, inhalation, and dermal contact, which pose non-carcinogenic and carcinogenic risks to human health.

(1) Exposure calculation

The daily average non-carcinogenic and carcinogenic PTE exposure pathways are calculated as Equations (6)–(8):

(6) $${ADD}_{iing}=\frac{C_{i}\times IngR\times EF\times ED}{BW\times AT}\times 10^{-6}$$

(7) $${ADD}_{iinh}=\frac{C_{i}\times InhR\times EF\times ED}{PEF\times BW\times AT}$$

(8) $${ADD}_{iderm}=\frac{C_{i}\times SA\times SL\times ABS\times EF\times ED}{BW\times AT}\times 10^{-6}$$

where *ADD_iing_*, *ADD_i_*_inh_, and *ADD_i_*_derm_ denote the average daily exposure of a PTE by oral ingestion, inhalation, and dermal contact, respectively, and *C_i_* denotes the concentration of a PTE (mg/kg). Parameters were taken from HJ 25.3 [46] and USEPA [45,47] (Table 4).

Children are more likely to be exposed to carcinogenic PTEs than adults. Exposure levels for children and adults need to be calculated separately, then as a weighted averaged, and finally assigned to the entire life cycle. The formula is as Equations (9)–(11):

(9) $${LADD}_{iing}=\frac{C_{i}\times EF}{AT}\left(\frac{{IngR}_{child}\times {ED}_{child}}{{BW}_{child}}+\frac{{IngR}_{adult}\times {ED}_{adult}}{{BW}_{adult}}\right)\times 10^{-6}$$

(10) $${LADD}_{iinh}=\frac{C_{i}\times EF}{PEF\times AT}\times \left(\frac{{InhR}_{child}\times {ED}_{child}}{{BW}_{child}}+\frac{{InhR}_{adult}\times {ED}_{adult}}{{BW}_{adult}}\right)$$

(11) $${LADD}_{iderm}=\frac{C_{i}\times EF\times SL\times ABS}{AT}\times \left(\frac{{SA}_{child}\times {ED}_{child}}{{BW}_{child}}+\frac{{SA}_{adult}\times {ED}_{adult}}{{BW}_{adult}}\right)\;\times 10^{-6}$$

(2) Risk characterization

Non-carcinogenic and carcinogenic risks arre assessed as Equations (12) and (13):

(12) $$HI=\sum_{i=1}^{n}HQ_{i}=\sum_{i=1}^{n}\frac{{ADD}_{iing}+{ADD}_{iinh}+{ADD}_{iderm}}{{RfD}_{i}}$$

(13) $$TCR=\sum_{i=1}^{n}CR_{i}=\sum_{i=1}^{n}({ADD}_{iing}+{ADD}_{iinh}+{ADD}_{iderm})\times {SF}_{i}$$

where *HI* is the index of non-carcinogenic risk of all PTEs; *HQ_i_* is the index of non-carcinogenic risk of a given PTE; *RfD_i_* is the non-carcinogenic average daily intake of a given PTE. *HI* or *HQ_i_* < 1 indicates that the non-carcinogenic risk can be ignored, otherwise, the risk cannot be ignored [47]. *CR* is the index of carcinogenic health risk of all PTEs, *CR_i_* refers to the index of carcinogenic risk of a particular PTE, and SF is the carcinogenic slope factor. The *RfD* and *SF* values for the exposure routes are shown in Table 5. The acceptable carcinogenic health risk index *TCR* or *CR_i_* ranges from 1 × 10^−6^ to 1 × 10^−4^, indicating an acceptable carcinogenic risk, whereas values above 1 × 10^−4^ indicate significant health hazards [48,49].

### 2.5. Statistical Analysis

Basic descriptive statistical analyses and box-normal plots were performed using the Origin 2022 (Origin Lab, Northampton, MA, USA). The ArcGIS 18.0 (ESRI, Redlands, CA, USA) was used for map delineation. *I_geo_*, *EF*, *NPI*, *PERI*, and *HI* were performed using Excel 365 (Microsoft Inc., Seattle, WA, USA).

## 3. Results and Discussion

### 3.1. Statistical Characteristics and Spatial Pattern of PTEs

The statistical summary of PTEs and pH are shown in Table 6. The soil pH ranged from 4.94 to 5.88, with a mean of 5.33 ± 0.10, and was predominantly acidic. Concentrations of As, Cr, Cu, Hg, Ni, Pb, and Zn in agricultural soil samples did not exceed the screening values of the risk control standard for environmental quality of soils on agricultural land [50], while the Cd concentration exceeded the screening value in only one sample. Compared with the Heilongjiang province background value [39], the average values of As, Cd, Hg, Pb, and Zn were higher than those (Table 2), while the average values of Cr, Cu, and Ni were lower than those; Pb and Cr were similar to those. The average concentration of As, Cd, Hg, Pb, Zn was 1.41, 1.22, 2.27, 1.02, and 1.22 times, while the concentration of Cr, Cu, and Ni was 0.97, 0.64, and 0.65, respectively.

The coefficient of variation reflected the homogeneity of the distribution of the element and the extent of variation, and also indicated whether it was influenced by multiple sources. The coefficients of variations of the 8 PTEs measured in the soil were in the following order: Cd (74.35%) > Cu (42.38%) > Ni (35.89%) > Zn (33.36%) > Hg (33.09%) > As (30.23%) > Cr (16.82%) > Pb (13.58%). Pb and Cr had a low variability; As, Hg, Ni and Zn had a moderate variability; and Cu and Cd had a high variability. The Cd had a coefficient of variation of 74.35%, indicating a possible influence of multiple sources [51].

**Figure 2:**
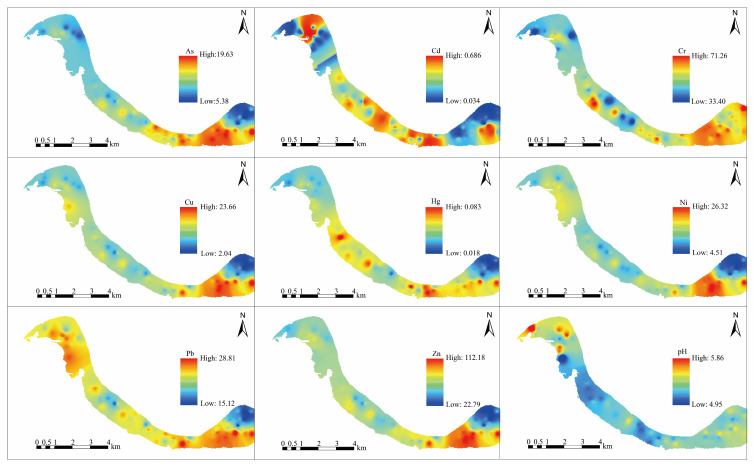
Spatial pattern of PTEs and pH.

The spatial pattern of PTEs and pH were derived from the spatial differences of the inverse distance weights of the PTEs (Figure 2). The lowest values of PTEs occured in the northeast part of the study area, which was an area of Holocene alluvium formation, except for Cr. The high values of As, Cr, Cu, Ni, and Zn appeared in the southeast part of the study area. The high values of Hg and Pb appeared as diffusion patterns in most of the area, with the highest values near residential areas. The highest values of Cd were found separately in the southeast, center, and northwest of the study area.

### 3.2. Source Apportionment of PTEs

Anthropogenic and geogenic/pedogenic inputs are often mixed, and both contribute to the presence of PTEs in soils [52]. Identifying sources of PTEs in remote permafrost agricultural ecosystems is crucial to evaluate the influence of geogenic and anthropogenic activities and to understand their biogeochemical processes. The correlations of PTEs are shown in Table 7. The high correlation coefficient among PTEs indicated that accumulated PTEs were formed from similar sources. There were significant correlations between As, Cu, Ni, and Zn (R > 0.877, *p* < 0.01). Hg and Pb were well-correlated (R = 0.715, *p* < 0.01). There were low correlations between Cd with other PTEs (R < 0.568, *p* < 0.01), except for Cr. Cr was not correlated with Cd, Hg, and Pb, and had a low correlation with As, Cu, Ni, and Zn (R < 0.399, *p* < 0.01).

**Figure 3:**
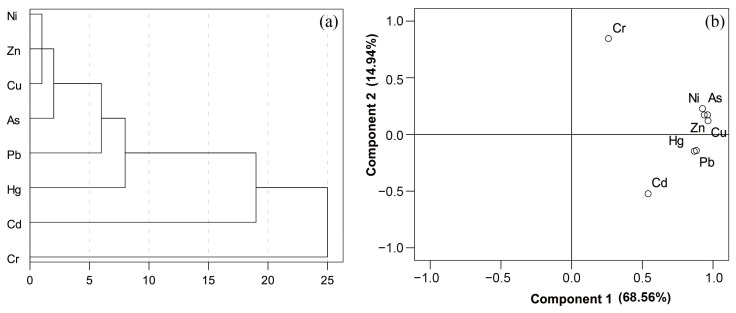
(**a**) CA and (**b**) PCA of PTEs in agricultural soils.

The principal component analysis (PCA) was carried out to identify similarities of PTEs in soils(Table 8). All soil data set passed the KMO and Barrett tests (KMO: 0.87, Barrett significance: 0.00). The factors were rotated by the maximum variance method, indicating no correlation between the extracted dimensions. There were two components of the loading plot of principal component analysis (68.56% and 14.94%) (Figure 3). The F1 was characterized by As, Cd, Cu, Hg, Ni, Pb, and Zn, contributing to the total variances (68.56%). The F2 was characterized by Cr, which described 14.94% of total variances.

The cluster analyses of PTEs were carried out according to the square Euclidean distance using the intergroup connection method (Figure 3). PTEs could be roughly divided into three or four categories. The first category was As, Cu, Ni, and Zn. The second type was Hg and Pb. The third type was Cd, and the fourth type was Cr. The cluster analysis was mainly consistent with the results of correlation analysis and principal component analysis, implying that As, Cu, Ni, and Zn may have originated from similar sources, and Hg and Pb may have originated from another source. While Cd and Cr were likely enriched by different mechanisms.

As-enriched rocks, such as black mudstone [53], were widely distributed in the south of the study area (Figure 1). Black mudstones were deposited in anoxic, stagnant aquatic environments, producing sediments rich in organic matter and sulphides. Arsenic is a strong chalcophile element and its occurrence is usually associated with that of arsenopyrite and other sulphide and sulphoarsenide compounds, such as copper, lead, zinc, and nickel in sulphide deposits [54,55]. It could be released into the environment during the weathering or mining process. Therefore, As, Cu, Zn and Ni were significantly positively associated and grouped into one category. Due to the cold air temperature of permafrost regions, atmospheric deposition rates in these regions were high, and various pollutants are settled and accumulated in these regions [21].

Lead was probably the most extensively investigated PTE. This was because Pb had been widely dispersed in the environment since the onset of metallurgy, and more recently had been used globally as an additive in gasoline. Mercury was another metal of particular environmental concern given its high volatility, long atmospheric residence time, and intrinsic toxicity [56]. Because of the “cold-trapping” effect, Hg and Pb could be transported from populated regions to high latitude or altitude regions by atmospheric circulation, and deposited to the soils [22,57]. Therefore, the Pb and Hg observed in agricultural areas in this study were closely related to long-range atmospheric transport.

Previous studies have shown that agricultural activities increase the use of chemical fertilizers, thus leading to the enrichment of Cd in topsoil [51,58]. Therefore, the different pattern of Cd observed in agricultural areas in this study were likely related to human planting activities.

Cr occurs naturally in ultramafic rocks and may persist in parent minerals, co-precipitated with manganese, aluminum, and/or iron oxides, and hydroxides, generally adsorbed on soil particles and complexed with soil organic compounds. High levels of Cr in the northeast of the study area may be caused by the different substrates of the Holocene alluvium (Figure 1).

### 3.3. Pollution Assessment of PTEs

Geo-accumulation index (*I_geo_*). Using the background value of surface soil in Heilongjiang Province as the evaluation standard, the degree of PTE pollution of agricultural soils in the study area was evaluated by the *I_geo_*. The values determined for As, Cd, Cr, Cu, Hg, Ni, Pb, and Zn at *I_geo_* ranged from −1.35 to 0.62 (mean −0.16), from −2.11 to 2.45 (mean −0.47), from −1.31 to −0.16 (mean −0.65), from −4.51 to −0.25 (mean −1.40), from −0.95 to 1.35 (mean 0.40), from −3.04 to −0.38 (mean −1.32), from −1.15 to −0.17 (mean −0.57), and from −2.01 to 0.43 (mean −0.40), respectively. The mean value of *I_geo_* of PTEs in descending order was Hg > As > Zn > Cd > Pb > Cr > Ni > Cu, where Hg was with 69.33% of the uncontaminated- to moderately-contaminated samples and 10.67% of the moderately contaminated samples, As with 37.33% of uncontaminated- to moderately-contaminated samples, Zn with 22.67% of uncontaminated- to moderately-contaminated samples, Cd with 12% of uncontaminated- to moderately-contaminated samples, but one sample with moderately- to strongly-contaminated. The *I_geo_* of Cr, Cu, Ni and Pb were generally not contaminated.

**Figure 4:**
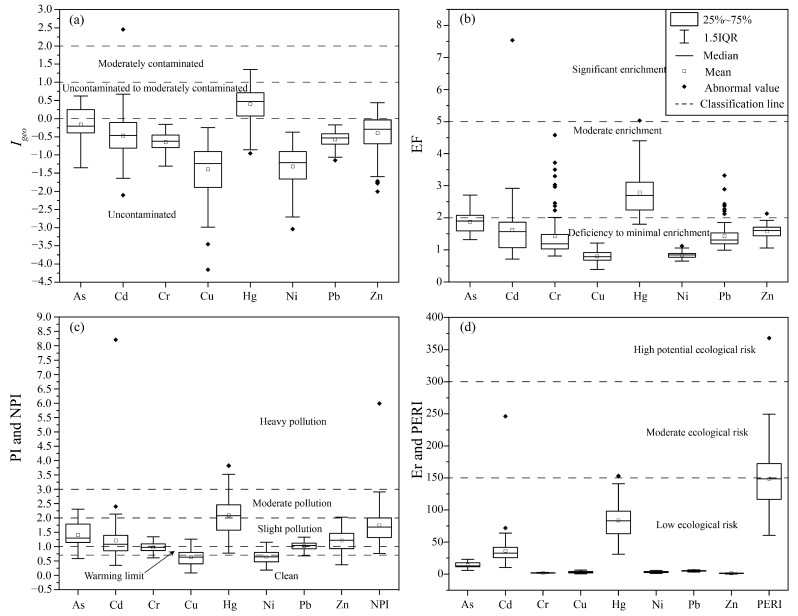
Box plots of (**a**) *I_geo_*, (**b**) *EF*, (**c**) *PI* and *NPI*, (**d**) *Er* and *PERI* for PTEs in soils.

Enrichment Factor (*EF*). The enrichment factor (*EF*) is useful for understanding the impact of anthropogenic activities on soil. *EF* values for PTEs are shown in Figure 4. The *EF* values for As, Cd, Cr, Cu, Hg, Ni, Pb, and Zn ranged from 1.32 to 2.71 (mean 1.88), from 0.71 to 7.54 (mean 1.62), from 0.81 to 4.58 (mean 1.43), from 0.39 to 1.21 (mean 0.80), from 1.80 to 5.03 (mean 2.79), from 0.65 to 1.12 (mean 0.83), from 0.99 to 3.32 (mean 1.43), and from 1.06 to 2.13 (mean 1.58), respectively. The mean values of *EF* were in the order of Hg > As > Cd > Zn >Cr = Pb > Ni > Cu. *EF* values of Cu and Ni in soils were less than 2, indicating deficiency to minimal enrichment. *EF* values of As in 64.00% and 36.00% of the soils were in the class of deficiency to minimal enrichment and moderate enrichment, respectively. For Cd, 81.00%, 17.00%, and 1.00% of the soils were in the class of deficiency to minimal enrichment, moderate enrichment, and significant enrichment, respectively. *EF* values for Cr in 87.00% and 13.00% of the soils were in the class of deficiency to minimal enrichment and moderate enrichment, respectively. *EF* values for Hg in 12.00%, 87.00%, and 1.00% of the soils were in the class of deficiency to minimal enrichment, moderate enrichment, and significant enrichment, respectively. The values of *EF* for Pb in 91.00% and 9.00% of the soils were in the class of deficiency to minimal enrichment and moderate enrichment. For Zn, on the other hand, 99% and 1% of the soils were in the class of deficiency to minimal enrichment and moderate enrichment.

Nemerow Pollution Index (*NPI*). The *PI* and *NPI* values for PTEs are shown in Figure 4. The values determined for As, Cd, Cr, Cu, Hg, Ni, Pb, and Zn at *PI* ranged from 0.59 to 2.31 (mean 1.41), from 0.35 to 8.20 (mean 1.21), from 0.61 to 1.34 (mean 0.97), from 0.08 to 1.26 (mean 0.64), from 0.77 to 3.83 (mean 2.09), from 0.18 to 1.16 (mean 0.65), from 0.68 to 1.33 (mean 1.02), and from 0.37 to 2.03 (mean 1.22), respectively. The mean values of *PI* were in the order Hg > As > Zn ≈ Cd > Pb > Cr >Ni ≈ Cu. The *PI* values of As in 4.00%, 14.67%, 69.33%, and 12.00% of the soils were in the class of clean, warning limit, slight pollution and moderate pollution, respectively. For Cd, 10.67%, 29.33%, 56%, 2.67%, and 1.33% of the soils were in the class of clean, warning limit, slight pollution, moderate pollution, and heavy pollution, respectively. For Cr, 8.00%, 50.67%, and 41.33% of the soils were in the class of clean, warning limit, and slight pollution, respectively. For Cu, 62.67%, 26.67%, and 10.67% of the soils were in the class of clean, warning limit, and slight pollution, respectively. For Hg, 8.00%, 38.67%, 42.67%, and 10.67% of the soils were in the class of warning limit, slight pollution, moderate pollution, and heavy pollution, respectively. For Ni, 61.33%, 28.00%, and 10.67% of the soils were in the class of clean, warning limit, and slight pollution, respectively. For Pb, 1.33%, 37.33%, and 61.33% of the soils were in the class of clean, warning limit, and slight pollution, respectively. For Zn, 9.33%, 22.67%, 66.67%, and 1.33% of the soils were in the class of clean, warning limit, slight pollution, and moderate pollution, respectively. The *NPI* values ranged from 0.76 to 5.99, with a mean value of 1.75. Additionally, 8%, 66.67%, 24%, and 1.33% of the soilsweare in the class of warning limit, slight pollution, moderate pollution, and heavy pollution, respectively.

**Figure 5:**
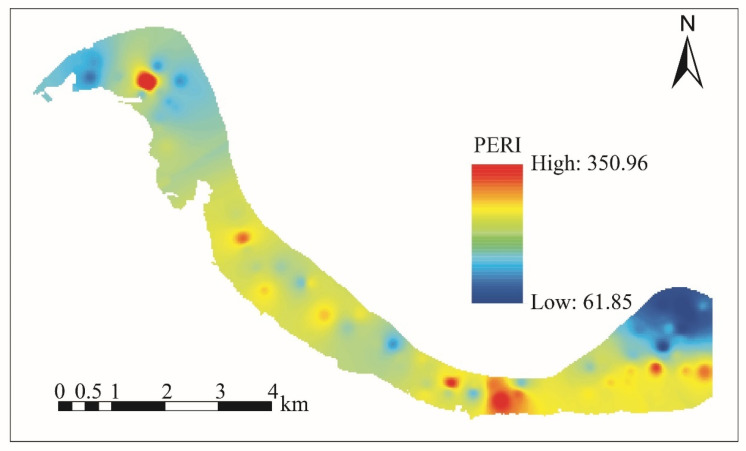
Spatial pattern of PERI.

Potential ecological risk (*PERI*). The *Er* values for As, Cd, Cr, Cu, Hg, Ni, Pb, and Zn ranged from 5.88 to 23.08 (mean 14.08), from 10.44 to 246.05 (mean 36.38), from 1.21 to 2.69 (mean 1.94), from 0.42 to 6.32 (mean 3.18), from 30.97 to 153.17 (mean 83.72), from 0.91 to 5.78 (mean 3.24), from 3.39 to 6.66 (mean 5.09), and from 0.37 to 2.03 (mean 1.22), respectively. The mean *Er* values were in the order Hg > Cd > As > Pb > Ni > Cu > Cr > Zn. The *Er* values of As, Cr, Cu, Ni, Pb, and Zn were all below 40, indicating a low ecological risk. The *Er* values of Cd showed a low ecological risk, moderate ecological risk, and high ecological risk in 72.00%, 26.67%, and 1.33% of soil samples, respectively. The *Er* of Hg showed a low ecological risk, moderate ecological risk, and considerable ecological risk in 8.00%, 38.67%, and 53.33% of soil samples, respectively. The *PERI* of all soil samples ranged from 60.31 to 367.83 (mean 148.85), and showed low ecological risk, moderate ecological risk, and considerable ecological risk in 50.67%, 48.00%, and 1.33%, respectively. Figure 5 shows that the considerable ecological risk is located in the northwestern part of the study area, while the moderate ecological risk is located in the southeastern part.

### 3.4. Human Health Risk Assessment of PTEs

Exposure of PTEs. The average daily exposure to PTEs are included in Table 9. They are in the order of direct oral ingestion > dermal exposure > inhalation. The average daily intake level for adults and children, in descending order, are *ADD_iing_* (*LADD_iing_*) > *ADD_iderm_* (*LADD_iderm_*) > *ADD_iinh_* (*LADD_iinh_*). The average daily non-carcinogenic exposures for three exposure pathways for PTEs in descending order are Zn > Cr > Pb > Ni > Cu > As > Cd > Hg. The mean daily exposure in a single metabolic pathway and the total daily exposure of children are higher than those of adults.

Non-carcinogenic risk. The list of values from *HQ* is included in Table 10. As can be seen from the table, the sum of PTE *HQ* values for the exposure routes of both subpopulations in the present study decreased in the order of *HQ_iing_* > *HQ_iderm_* > *HQ_iinh_*, except for Hg and Ni. This showed that ingestion was the predominant exposure route for PTEs affecting human health, followed by inhalation and skin contact, which was the least. These results were also reported by previous studies [59]. The non-carcinogenic risks of PTEs in adults and children were in the order of As > Cr > Pb > Ni > Hg > Cu > Zn > Cd. The mean value of individual PTE non-carcinogenic risk index was less than 1, which meanst that individual PTEs in agricultural soils in the study area did not pose any non-carcinogenic risk to human health yet.

The values for adults’ *HI* ranged from 0.068 to 0.164 with a mean of 0.112, which was less than the value of 0.124 in the cold black soil region [60], while more than the value of 0.105 was in the soil around the Qinghai Lake in Tibet Plateau [61] and 0.000353 in soil of landfill and geothermal sites in Tibet [62]. The values for children’s *HI* ranged from 0.188 to 0.453, with a mean of 0.310, which was less than the value of 0.839 [60], while more than the value of 0.185 [61] and 0.000383 [62]. For both adults and children, *HI* was less than 1, indicating that the non-carcinogenic health risks to adults and children from PTEs in the agricultural soils of the study area were low.

**Figure 6:**
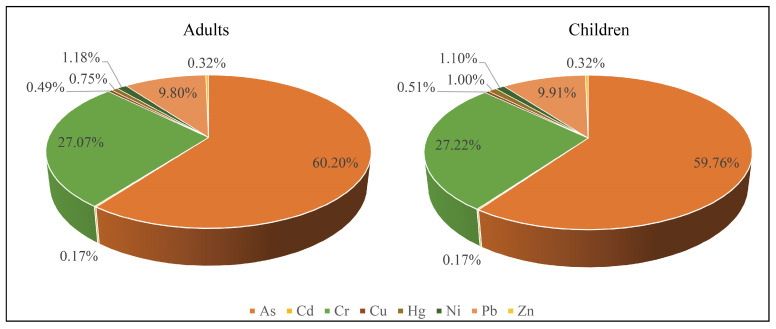
Adults and children *HQ* contribution rate of PTEs in the soil.

As Figure 6 shows, the major non-carcinogenic factors of PTEs in agricultural soils were As, Cr, and Pb, with the sum of the three elements accounting for more than 95% of *HQ*.

Carcinogenic risk. Since slope factors were currently available for only two elements, As and Cd, only the carcinogenic risk of As and Cd exposure in agricultural soils was evaluated. As for *HI*, the *CR* shows *CR*_iing_ > *CR_iderm_* > *CR_iinh_*, suggesting that the oral intake route was the main factor for the carcinogenic risk.

The results showed that the *CR_adults_* ranged from 4.30 × 10^−6^ to 1.65 × 10^−5^, with a mean of 1.02 × 10^−5^, which was less than the value of 1.21 × 10^−5^ [61], while more than the value of 9.97 × 10^−6^ [60] and 6.59 × 10^−8^ [62]. The *CR_children_* ranged from 1.18 × 10^−5^ to 4.55 × 10^−5^, with a mean value of 2.82 × 10^−5^, which was more than the value of 2.68 × 10^−5^ [60], 2.15 × 10^−5^ [61], 1.83 × 10^−8^ [62]. The *CR_adults_* and *CR_children_* values of all samples were within the acceptable range of 10^−6^ to 10^−4^, indicating that there was no significant carcinogenic health risk to the local adults and children.

## 4. Conclusions

The average concentration (mean ± standard deviation) of As, Cd, Cr, Cu, Hg, Ni, Pb, and Zn were 12.11 ± 3.66 mg/kg, 0.11 ± 0.08 mg/kg, 52.50 ± 8.83 mg/kg, 12.08 ± 5.12 mg/kg, 0.05 ± 0.02 mg/kg, 14.90 ± 5.35 mg/kg, 22.38 ± 3.04 mg/kg, and 68.07 ± 22.71 mg/kg, respectively. The average concentration of Hg, As, Cd, and Zn in agricultural soils in the study area were 2.09, 1.41, 1.21, and 1.22 times higher than the Heilongjiang background values, respectively; the average concentration of Pb and Cr were comparable to the background values, and the average concentration of Cu and Ni were significantly lower than the background values.

The variation of the coefficients was in the following order: Cd (74.35%) > Cu (42.38%) > Ni (35.89%) > Zn (33.36%) > Hg (33.09%) > As (30.23%) > Cr (16.82%) > Pb (13.58%). With the exception of Cr and Pb, the remaining six elements exhibited moderate-to-high variability and may be influenced by multiple sources.

The correlation between PTEs was significant (*p* < 0.01 and *p* < 0.05), except for Cr with Cd, Hg, and Pb, respectively. The results of CA and PCA indicated that As, Cu, Ni and Zn were likely to have originated from geogenic/pedogenic processes, Hg and Pb were likely to have originated from long-range atmospheric transport, while Cd and Cr were likely to have originated from both natural and anthropogenic sources.

The *I_geo_* and *EF* showed that As, Cd, Hg, and Zn were enriched in soils. The *I_geo_* of Hg showed that 80% of the samples reached moderately contaminated and moderately to strongly contaminated levels. The *I_geo_* of Cd showed that 12% of the samples reached the level uncontaminated to moderately contaminated, but one sample reached the level moderately to strongly contaminated. The *I_geo_* of As and Zn, 37.33% and 22.67%, of the samples reached the level uncontaminated to moderately contaminated, respectively. The remaining PTEs were free of contamination. The mean values of *EF* were in descending order: Hg > As > Cd > Zn > Cr = Pb > Ni > Cu, with 86.67%, 36,% and 17.33% of the samples showing moderate enrichment and above with Hg, As, and Cd, respectively.

The *NPI* ranged from 0.76 to 5.99, with the mean 1.75, showing that 66.67%, 24%, and 1.33% of soil samples in slight, moderate and heavy pollution levels, respectively, with Hg being the most important element affecting the comprehensive pollution index. The *PERI* ranged from 60.31 to 367.83, with 48.00% and 1.33% of the soil samples in the moderate ecological risk and high potential ecological risk, respectively.

The non-carcinogenic *HIs* for adults and children were less than 1, which was within the acceptable range. In addition, the carcinogenic risks to adults and children were within acceptable range.

## Figures and Tables

**Table 1:** Detection limits (DL) required of the study.

Element	Unit	Recommended Detection Limit	Analytical Method	Digestion Method
As	mg/kg	1	AFS	aqua regia
Cd	mg/kg	0.03	ICP-MS	HF+HCl+HNO_3_+HClO_4_
Cr	mg/kg	5	XRF	Pressed powder pellets
Cu	mg/kg	1	XRF	Pressed powder pellets
Hg	mg/kg	0.0005	AFS	aqua regia
Ni	mg/kg	2	XRF	Pressed powder pellets
Pb	mg/kg	2	XRF	Pressed powder pellets
Zn	mg/kg	4	XRF	Pressed powder pellets
Sc	mg/kg	1	ICP-OES	HF+HCl+HNO_3_+HClO_4_
pH		0.1	ISE	

AFS: atomic fluorescence spectrometry, Haiguang Instrument Co. Ltd., Beijing, China; ICP-MS: inductively coupled plasma-mass spectrometry, Thermo Fisher Scientific, Waltham, MA, USA; XRF: X-ray fluorescence spectrometry, PANalytical, Almelo, The Netherlands; ICP-OES: inductively coupled plasma-optical emission spectrometry, Thermo Fisher Scientific, Waltham, MA, USA; ISE: ion selective electrode, Metrohm, Herisau, Switzerland.

**Table 2:** Allowance of accuracy and precision for routine analysis.

Concentration Range	Accuracy	Precision
	$\overset{-}{\Delta lgC}=\left|lg\overset{-}{C_{i}}-lgC_{s}\right|$	$\lambda =\sqrt{\frac{\sum_{i=1}^{4}{\left(lgC_{i}-lgC_{s}\right)}^{2}}{4-1}}$
<3 detection limit	≤0.12	0.20
>3 detection limit	≤0.10	0.17
1–5%	≤0.07	0.15
>5%	≤0.05	0.08

$\overset{-}{C_{i}}$, the average determined value of ${SRM}_{i}$; $C_{i}$, the determined value of ${SRM}_{i}$; $C_{s}$, the recommended value of ${SRM}_{i}$.

**Table 3:** Classes of indices: *I_geo_*, *EF*, *NPI*, *Er*, and *PERI*.

Class	*I_geo_*	*EF*	*NPI*	*E_r_*	*PERI*
1	≤0, uncontaminated	<2, deficiency to minimal enrichment	≤0.7, clean	<40, low ecological risk	<150, low ecological risk
2	0–1, uncontaminated to moderately contaminated	2–5, moderate enrichment	0.7–1, warning limit	40–80, moderate ecological risk	150–300, moderate ecological risk
3	1–2, moderately contaminated	5–20, significant enrichment	1–2, slight pollution	80–160, considerable ecological risk	300–600, high potential ecological risk
4	2–3, moderately to strongly contaminated	20–40, very high enrichment	2–3, moderate pollution	160–320, high ecological risk	≥600, significantly high ecological risk
5	3–4, strongly contaminated	>40, extremely high enrichment	>3, heavy pollution	≥320, serious ecological risk	
6	4–5, strongly to extremely contaminated				
7	>5, extremely high contaminated				

Geo-accumulation index (*I_geo_*), enrichment factor (*EF*), Nemerow Pollution Index (*NPI*), Single Index of Ecological Risk (*Er*), Potential Ecological Risk Index (*PERI*).

**Table 4:** Health risk exposure parameters of PTEs.

Symbol	Parameter	Unit	Adult Reference Value	Child Reference Value
*ED*	Exposure Years	a	24	6
*BW*	Average Weight	kg	61.8	19.2
*EF*	Exposure Frequency	d/a	350	350
*AT*	Average Exposure Time	d	Carcinogenic27740 Noncarcinogenic9125	Carcinogenic27740 Noncarcinogenic9125
*IngR*	Daily Soil Intake	mg/d	100	200
*InhR*	Daily Air Respiration	m^3^/d	14.5	7.5
*SA*	Exposed Skin Surface Area	cm^2^	5373.99	2848.01
*SL*	Skin Adhesion Coefficient	mg/(cm^2^·d)	0.07	0.2
*PEF*	Surface Dust Emission Factor	m^3^/kg	1.36 × 10^9^	1.36 × 10^9^
*ABS*	Skin Absorption Factor		As: 0.03; Cd: 0.001; Cr:0.001; Cu: 0.06; Hg: 0.05; Ni: 0.001; Pb: 0.006; Zn: 0.02	

**Table 5:** PTEs reference measurement and carcinogenic slope factor.

PTEs	Reference Measurement *RfD* [mg/(kg·d)]				Carcinogen *SF* [(kg·d)/mg]		
	*ADD_iing_*	*ADD_iderm_*	*ADD_iinh_*	*LADD_iinh_*	Through Mouth	Skin	Breathing
As	3.0 × 10^−4^	3.0 × 10^−4^	3.52 × 10^−6^	5.86 × 10^−6^	1.5	1.5	4.3 × 10^−3^
Cd	1.0 × 10^−3^	2.5 × 10^−5^	2.35 × 10^−6^	3.91 × 10^−6^	6.1	6.1	6.3
Cr	3.0 × 10^−3^	7.5 × 10^−5^	2.35 × 10^−5^	3.91 × 10^−5^			
Cu	4.0 × 10^−2^	4.0 × 10^−2^					
Hg	3.0 × 10^−4^	2.1 × 10^−5^	7.04 × 10^−5^	1.17 × 10^−5^			
Ni	2.0 × 10^−2^	8.0 × 10^−4^	2.11 × 10^−5^	3.52 × 10^−5^			
Pb	3.5 × 10^−3^	5.3 × 10^−4^	8.21 × 10^−5^	1.37 × 10^−4^			
Zn	3.0 × 10^−1^	3.0 × 10^−1^					

**Table 6:** Statistical summary of PTEs concentrations (mg/kg) and pH in soil samples.

PTE	As	Cd	Cr	Cu	Hg	Ni	Pb	Zn	pH
Minimum Value	5.06	0.031	32.70	1.60	0.017	4.20	14.90	20.90	4.94
Maximum Value	19.85	0.738	72.60	24.00	0.084	26.60	29.30	113.50	5.88
Median Value	11.17	0.10	52.7	12.10	0.05	14.90	22.80	68.50	5.31
Mean Value	12.11	0.11	52.50	12.08	0.05	14.90	22.38	68.07	5.33
Standard Deviation	3.66	0.08	8.83	5.12	0.02	5.35	3.04	22.71	0.10
Coefficients of Variation	30.23%	74.35%	16.82%	42.38%	33.09%	35.89%	13.58%	33.36%	1.93%
Screening Values [50]	40.00	0.30	150.00	50.00	1.30	60.00	70.00	200.00	-
Background Values [39]	8.60	0.090	54.00	19.00	0.022	23.00	22.00	56.00	8.3

**Table 7:** Linear relationship coefficients between each PTE in the agricultural soils.

	As	Cd	Cr	Cu	Hg	Ni	Pb	Zn
As	1	0.485 **	0.399 **	0.899 **	0.774 **	0.877 **	0.743 **	0.932 **
Cd		1	−0.034	0.410 **	0.568 **	0.294 *	0.430 **	0.433 **
Cr			1	0.289 *	0.159	0.321 **	0.028	0.347 **
Cu				1	0.788 **	0.955 **	0.853 **	0.954 **
Hg					1	0.700 **	0.715 **	0.764 **
Ni						1	0.818 **	0.960 **
Pb							1	0.806 **
Zn								1

* *p* < 0.05. ** *p* < 0.01.

**Table 8:** Factor loadings of components and those obtained after matrix rotation.

PTEs	Component Matrix		Rotated Component Matrix	
	PC1	PC2	PC1	PC2
As	0.948	0.099	0.938	0.172
Cd	0.499	−0.563	0.541	−0.523
Cr	0.325	0.824	0.26	0.847
Cu	0.971	0.045	0.965	0.121
Hg	0.853	−0.215	0.867	−0.148
Ni	0.94	0.155	0.925	0.228
Pb	0.869	−0.21	0.882	−0.142
Zn	0.972	0.097	0.961	0.173

Extraction method: principal component analysis; rotation method: Varimax with Kaiser normalization.

**Table 9:** Average daily exposure of PTEs to non-carcinogenic in soils [mg/(kg·d)].

PTEs	Adult				Child			
	*ADD_iing_*	*ADD_iderm_*	*ADD_iinh_*	*ADD_adult_*	*LADD_iing_*	*LADD_iderm_*	*LADD_iinh_*	*LADD_child_*
As	1.80 × 10^−5^	2.04 × 10^−6^	1.92 × 10^−9^	2.01 × 10^−5^	4.70 × 10^−5^	8.29 × 10^−6^	2.72 × 10^−9^	5.54 × 10^−5^
Cd	1.63 × 10^−7^	6.12 × 10^−10^	1.73 × 10^−11^	1.63 × 10^−7^	4.24 × 10^−7^	2.49 × 10^−9^	2.45 × 10^−11^	4.27 × 10^−7^
Cr	7.82 × 10^−5^	2.94 × 10^−7^	8.34 × 10^−9^	7.85 × 10^−5^	2.04 × 10^−4^	1.20 × 10^−6^	1.18 × 10^−8^	2.05 × 10^−4^
Cu	1.80 × 10^−5^	4.06 × 10^−6^	1.92 × 10^−9^	2.21 × 10^−5^	4.69 × 10^−5^	1.65 × 10^−5^	2.72 × 10^−9^	6.34 × 10^−5^
Hg	6.86 × 10^−8^	1.29 × 10^−8^	7.31 × 10^−12^	8.15 × 10^−8^	1.79 × 10^−7^	5.26 × 10^−8^	1.04 × 10^−11^	2.32 × 10^−7^
Ni	2.22 × 10^−5^	8.35 × 10^−8^	2.37 × 10^−9^	2.23 × 10^−5^	5.79 × 10^−5^	3.40 × 10^−7^	3.35 × 10^−9^	5.83 × 10^−5^
Pb	3.33 × 10^−5^	7.53 × 10^−7^	3.55 × 10^−9^	3.41 × 10^−5^	8.70 × 10^−5^	3.07 × 10^−6^	5.03 × 10^−9^	9.01 × 10^−5^
Zn	1.01 × 10^−4^	7.63 × 10^−6^	1.08 × 10^−8^	1.09 × 10^−4^	2.65 × 10^−4^	3.11 × 10^−5^	1.53 × 10^−8^	2.96 × 10^−4^
∑	2.71 × 10^−4^	1.48 × 10^−5^	2.89 × 10^−8^	2.86 × 10^−4^	7.08 × 10^−4^	6.06 × 10^−5^	4.10 × 10^−8^	7.69 × 10^−4^

**Table 10:** The mean value of non-carcinogenic health risk index of PTEs in soils.

PTEs	Adults				Children			
	*HQ_iing_*	*HQ_iderm_*	*HQ_iinh_*	*HQ*	*HQ_iing_*	*HQ_iderm_*	*HQ_iinh_*	*HQ*
As	6.01 × 10^−2^	6.79 × 10^−3^	5.47 × 10^−4^	6.75 × 10^−2^	1.57 × 10^−1^	2.77 × 10^−2^	4.65 × 10^−4^	1.85 × 10^−1^
Cd	1.63 × 10^−4^	2.45 × 10^−5^	7.38 × 10^−6^	1.94 × 10^−4^	4.24 × 10^−4^	9.97 × 10^−5^	6.28 × 10^−6^	5.30 × 10^−4^
Cr	2.61 × 10^−2^	3.92 × 10^−3^	3.55 × 10^−4^	3.03 × 10^−2^	6.80 × 10^−2^	1.60 × 10^−2^	3.02 × 10^−4^	8.43 × 10^−2^
Cu	4.50 × 10^−4^	1.02 × 10^−4^		5.51 × 10^−4^	1.17 × 10^−3^	4.14 × 10^−4^		1.59 × 10^−3^
Hg	2.29 × 10^−4^	6.14 × 10^−4^	1.04 × 10^−7^	8.43 × 10^−4^	5.97 × 10^−4^	2.50 × 10^−3^	8.85 × 10^−7^	3.10 × 10^−3^
Ni	1.11 × 10^−3^	1.04 × 10^−4^	1.12 × 10^−4^	1.33 × 10^−3^	2.90 × 10^−3^	4.25 × 10^−4^	9.52 × 10^−5^	3.42 × 10^−3^
Pb	9.53 × 10^−3^	1.42 × 10^−3^	4.33 × 10^−5^	1.10 × 10^−2^	2.49 × 10^−2^	5.79 × 10^−3^	3.67 × 10^−5^	3.07 × 10^−2^
Zn	3.38 × 10^−4^	2.54 × 10^−5^		3.63 × 10^−4^	8.82 × 10^−4^	1.04 × 10^−4^		9.86 × 10^−4^
∑	9.80 × 10^−2^	1.30 × 10^−2^	1.06 × 10^−3^	1.12 × 10^−1^	6.80 × 10^−2^	5.30 × 10^−2^	9.06 × 10^−4^	3.10 × 10^−1^

## Data Availability

Data is unavailable due to privacy restrictions.
